# Bringing clinical and fundamental young microbiologists together

**DOI:** 10.1093/femsmc/xtac025

**Published:** 2022-10-16

**Authors:** Gaëtan Ligat, Théo Ghelfenstein-Ferreira, Sarah Dellière, Maxime Pichon

**Affiliations:** Institut Toulousain des Maladies Infectieuses et Inflammatoires (Infinity), INSERM, CNRS, UPS, Université de Toulouse, Toulouse, France; Assistance Publiques-Hôpitaux de Paris (AP-HP), Université de Paris,, Paris, France; Laboratoire de Parasitologie-Mycologie, AP-HP, Hôpital Saint-Louis, Paris, France; Institut Pasteur, CNRS, Unité de Mycologie Moléculaire, Université de Paris Cité, UMR2000, F-75015 Paris, France; CHU de Poitiers, Département des Agents Infectieux, Laboratoire de Bactériologie et Hygiène Hospitalière, Poitiers, France; Inserm, Pharmacologie des Agents Anti Infectieux et Antibiorésistance UMRS 1070, Université de Poitiers,, Poitiers, France

In the French Society for Microbiology (SFM), which is a member of the Federation of European Microbiological Societies (FEMS), interaction between young fundamental and clinical microbiologists has been promoted. Young microbiologists may have difficulties finding and obtaining grants, fellowships, and permanent positions. Recently, Dellière et al. (2021) explained how networking could help to resolve these problems. However, even though interactions and collaborations between researchers from diversified backgrounds are crucial, young microbiologists from different disciplines, in both medical and basic microbiology, are often isolated in their self-contained communities (Dellière et al. 2021). For these reasons, the creation of a section for young scientists has become essential. It is with the common goals of sharing experience, disseminating ideas, and providing training assistance that young French microbiologists from different backgrounds created the young microbiologists section of the SFM in 2018 as a dynamic and informal space in which young scientists could interact (Fig. 1).

**Figure 1.**
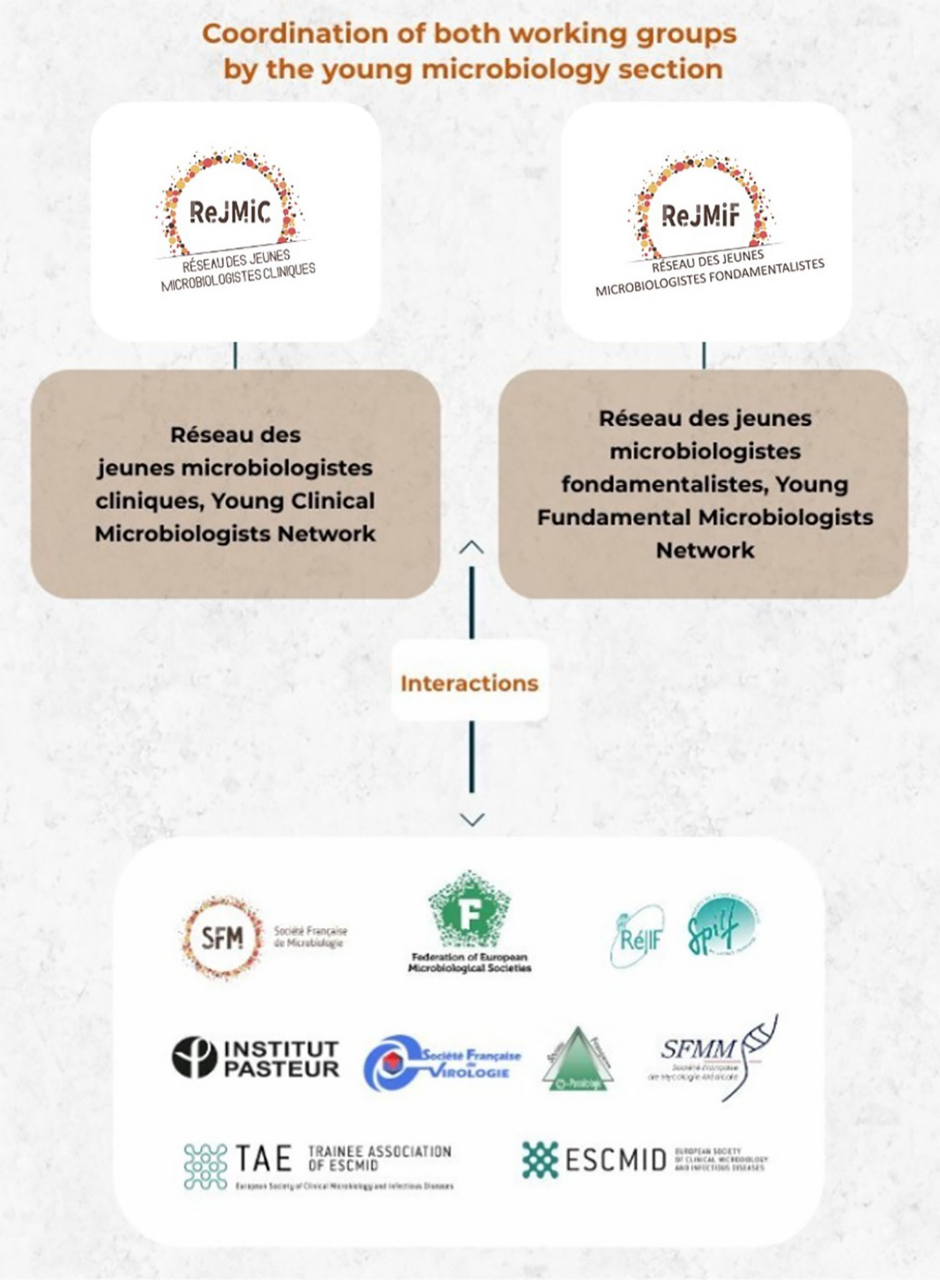
French young microbiology section organization. The Young Microbiologists section for the French SFM, was created in 2018 by young microbiologists as a dynamic and informal space for young scientists. The section is coordinated by two or three young scientists involved in fundamental and medical biology. The Young Microbiologists section successively structured two working groups: ReJMiC (Réseau des jeunes microbiologistes cliniques, Young Clinical Microbiologists Network) and ReJMiF (Réseau des jeunes microbiologistes fondamentalistes, Young Fundamental Microbiologists Network). Both groups are coordinated by the Young Microbiologists section and stimulate interactions between young trainees and with nonprofit scientific national organizations—such as the French Society of Virology, the French Society of Medical Mycology, or the French Infectious Diseases Society—or international organizations—such as the European Society of Clinical Microbiology and Infectious Diseases (ESCMID) or the FEMS.

The members of the section are highly engaged in clinical or fundamental microbiology involving various disciplines and in promoting all fields and aspects of microbiology. The Young Microbiologists section was one of the first groups of the SFM to conduct live thematic webinars with a keynote address (from a highly qualified senior in a specific field of research) and two short oral communications given by young scientists (based on their scientific quality, and evaluated by a jury constituted of young microbiologists). The success of the first webinar enabled us to create several live webinars, each of which was organized by a team of clinical and fundamental microbiologists. Furthermore, during the SFM congress, the Young Microbiologists section is organizing a career forum on Microbiology dedicated to young microbiologists. Different themes (thesis organization, mobility…) are discussed with senior microbiologists. In addition, the Young Microbiologists section has set up a Thesis prize and a Poster prize with senior scientists of the SFM, the objective being to highlight the work of young scientists. As planned, social media such as Twitter (for promoting social events, meetings, or sharing job or training opportunities; 2–twitter), Facebook (3–facebook), Instagram (for various quizzes; 4–instagram); and Linkedin (for sharing job or stage opportunities; 5–linkedIn) were successfully invested. Beyond bringing together young microbiologists from different horizons, we are also in contact with nonprofit international scientific organizations such as the European Society of Clinical Microbiology and Infectious Diseases (ESCMID; Dellière et al. 2021).

To satisfy the wide range of expectations and to facilitate structural organization two working groups have been created: the ReJMiC (Réseau des Jeunes Microbiologistes Cliniques, Young Clinical Microbiologists Network) and the ReJMiF (Réseau des Jeunes Microbiologistes Fondamentalistes, Young Fundamental Microbiologists Network). As their expectations are not completely the same (internship rotation jobs for clinical microbiologists; doctoral and postdoctoral positions for fundamental microbiologists), it was necessary to develop two working groups. Both of them propose personalized projects such as antimicrobial stewardship, games, events, and internship rotation job descriptions for the ReJMiC, and assistant professor or doctoral, postdoctoral fellowship applications, descriptions, and interviews of young microbiologists for the ReJMiF. Both working groups also work with other sections from the French SFM.

The ReJMiC actively collaborates with other networks of young infectious diseases professionals in France and in Europe. The common projects are articulated around two main axes, networking (encouraging exchanges between young people from different clinical specialties) and the promotion of young infectious diseases professionals among their peers.

- For the first axis, networking axis, the ReJMiC organized several receptions with the aim of bringing together young clinical microbiologists and young clinical infectiologists of the ReJIF (Réseau des Jeunes Infectiologues Français, Network of young French Infectiologists). Among these receptions, the event “serious game” combined pedagogy and networking. During informal events as previously cited, young people from different backgrounds can meet and get to know each other, thus creating their first professional network. In addition, to encourage contacts, the ReJMiC and the other young networks [ReJIF, Trainee association of ESCMID, JePPRI (Jeunes Professionnels de la Prévention du Risque Infectieux, Young Professionals in Infectious Risk Prevention)] invite each other to their respective congress. Finally, it is important for young infectious disease specialists to learn about the work of their clinical microbiology colleagues during their studies. To this end, ReJMiC has created a list of available microbiology laboratories that can accommodate young infectious disease specialists.
- The second axis concerns the promotion of young clinical microbiologists among their peers. To this end, ReJMiC is actively working on organizing conference sessions specifically for young people, in collaboration with the ReJIF.

The COVID-19 pandemic erupted only a year and a half after the still fragile young microbiologist section was created. Both research and networking were put on hold except for COVID-19 projects, while the clinical microbiologists among us were requisitioned for COVID-19 diagnosis. With working restrictions and complete or partial laboratory shutdowns worldwide, the COVID-19 pandemic impacted ongoing scientific projects, including scientific experiments (especially for Master and PhD student and postdoctoral projects with short-term contracts). However, scientific webinars, workshops, or meetings with international scientists can be facilitated by video-conferencing, which increased during the health crisis. Described above, a live thematic webinar of the Young Microbiologists section for the SFM was developed during the COVID-19 pandemic and allowed us to continue to work together and share projects and results. Since then, this model has been extensively applied by other work groups and sections in the SFM.

In 2021, Dellière et al. (2021) published a collaborative opinion paper between the Trainee Association of the ESCMID and four national trainee networks including the Young Microbiologists section. They discuss their motivations for building networks, the importance of these networks, and offer guidance for their creation and sustainability based on their extensive experience (defining the target audience and the objectives of the network, approaching national societies even in specific disciplines, built a steering committee, communications…; Dellière et al. 2021). Beyond the challenge of networking creation, this commentary emphasizes the benefits of interaction between young fundamental and clinical microbiologists in a scientific world in perpetual modification. We have shared our experience of the creation of the French Young Microbiologists networks, hosted by the SFM, and laid special emphasis on the challenges faced and the benefits reaped during the COVID-19 pandemic. To conclude, we hope that our commentary will encourage young microbiologists from other societies, and other countries, to take a similar approach to highlight young microbiologists' work and help them develop a perennial network essential to promising young researchers for a valuable service of science.

## Supplementary Material

xtac025_Supplemental_File
