# Supplementary material for: Bringing clinical and fundamental young microbiologists together
Source: FEMS Microbes. 2022 Oct 16;3:xtac025. doi: 10.1093/femsmc/xtac025 (PMC10117768; doi:10.1093/femsmc/xtac025)
Supplement: xtac025_Supplemental_File [file xtac025_supplemental_file.docx]

**Reviews of manuscript FEMSMC-2022-007**

**Reviewer 1**

**Recommendation: Minor Revision**

The commentary is clear and concise, and describes the creation of the French Young Microbiologists network. It argues convincingly for the integration of young members from both clinical and basic fields of microbiology to facilitate professional network building as well as potential cross-discipline projects. The authors have performed a valuable service to their peers and their field!

My hope is that this commentary will encourage young microbiologists from other societies, and other countries, to take a similar approach. To that end, I think it would be great for the authors to add a paragraph where they broaden their view a little bit, and make such a suggestion!

Also, for a commentary, an abstract is not necessary

Minor grammatical suggestions:

Line 82: remove 'society' from the end of the sentence

Line 91: therebefore? replace with 'above'?

Line 134: insert 'organizations' after 'national'

**Reviewer 2**

**Recommendation: Minor Revision**

This commentary has the exciting title of Bringing clinical and fundamental young microbiologists together. And what an outstanding platform for early career networking, especially during a very difficult period of isolation. However, I was hoping to read more concrete examples of outcomes that arose from bringing these two groups of young microbiologists together. For example, have any of these networking events resulted in new cross-disciplinary collaborations, manuscripts, grants, training opportunities, etc? I appreciate that it may be too soon to have data on such things, given that the group was only formed in 2018. In that case, were there specific activities working toward any of these goals. What would a 10 year successful young microbiologist chapter look like in terms of tangible outcomes? Do you think this should be expanded to other Microbiology Societies, if it's not already (is it?)? What lessons would you share/advice would you give/goals would you suggest to leaders of new chapters?

**Author's Response to Decision Letter for (FEMSMC-2022-007)**

Point by point response FEMSMC-2022-007

General comments to the Editor and Reviewer

We would like to thank the associate editor and the reviewers for giving us the opportunity to revise our manuscript. We highly appreciated their comments, which allowed us to improve our manuscript. We added two sections (page 3, line 83 and page 4, line 118). As suggested, we also removed the abstract and modified grammatical issues.

Response to Associate Editor

The commentary focuses on an important issue for early career researchers, on establishing a strong scientific network and collaborations to establish their career. The benefits of creating a "Young Microbiologists Section" in the French Microbiology society to manage the challenges due to the COVID pandemic were clear. However, for the commentary to be interesting to a broader audience, the article should present some tangible features as well as include reference to other young microbiology societies established in FEMS. Addressing the reviewers comments will strengthen this aspect of the commentary.

We thank the associate editor for these comments. We added a section and a sentence at the end of the commentary.

1. Page 3, line 83:

“The ReJMiC actively collaborates with other networks of young infectious diseases professionals in France and in Europe. The common projects are articulated around two main axes, networking (encouraging exchanges between young people from different clinical specialties) and the promotion of young infectious diseases professionals among their peers.

(i) For the first axis, networking axis, the ReJMiC organized several receptions with the aim of bringing together young clinical microbiologists and young clinical infectiologists of the ReJIF (Réseau des Jeunes Infectiologues Français, Network of young French infectiologists). Among these receptions, the event "serious game" combining pedagogy and networking (Ghelfenstein-Ferreira et al. 2021). During informal events as previously cited, young people from different backgrounds can meet and get to know each other, thus creating their first professional network. In addition, to encourage contacts, the ReJMiC and the other young networks (ReJIF, Trainee association of ESCMID, JePPRI [Jeunes Professionnels de la Prévention du Risque Infectieux, Young Professionals in Infectious Risk Prevention]) invite each other to their respective congress. Finally, it is important for young infectious disease specialists to learn about the work of their clinical microbiology colleagues during their studies. To this end, ReJMiC has created a list of available microbiology laboratory that can accommodate young infectious disease specialists.

(ii) The second axis concerns the promotion of young clinical microbiologists among their peers. To this end, ReJMiC is actively working on organizing conference sessions specifically for young people, in collaboration with the ReJIF.”

2. Page 4, line 118:

“In 2021, Dellière and colleagues published a collaborative opinion paper between the Trainee Association of European Society of Clinical Microbiology and Infectious Diseases (ESCMID) and four national trainee networks including the Young Microbiologists section. They discuss their motivations for building networks, the importance of these networks and offer guidance for their creation and sustainability based on their extensive experience (defining the target audience and the objectives of the network, approaching national societies even in specific disciplines, built a steering committee, communications…) (1). Beyond the challenge of networking creation, this commentary emphasizes the benefits of interaction between young fundamental and clinical microbiologists in a scientific world in perpetual modification. To conclude, we have shared our experience of the creation of the French Young Microbiologists networks, hosted by the SFM, and laid special emphasis on the challenges faced and the benefits reaped during the COVID-19 pandemic. To conclude, we hope that our commentary will encourage young microbiologists from other societies, and other countries, to take a similar approach to highlight young microbiologists work and help them develop a perennial network essential to promising young researchers for a valuable service of science.”

Response to Reviewer 1

The commentary is clear and concise, and describes the creation of the French Young Microbiologists network. It argues convincingly for the integration of young members from both clinical and basic fields of microbiology to facilitate professional network building as well as potential cross-discipline projects. The authors have performed a valuable service to their peers and their field! My hope is that this commentary will encourage young microbiologists from other societies, and other countries, to take a similar approach. To that end, I think it would be great for the authors to add a paragraph where they broaden their view a little bit, and make such a suggestion! Also, for a commentary, an abstract is not necessary.

Minor grammatical suggestions:

Line 82: remove 'society' from the end of the sentence

Line 91: therebefore? replace with 'above'?

Line 134: insert 'organizations' after 'national'

We thank the reviewer 1 for these comments and suggestions. We added two sections (see above). We also removed the abstract and modified grammatical suggestions.

1. Page 3, line 83:

“The ReJMiC actively collaborates with other networks of young infectious diseases professionals in France and in Europe. The common projects are articulated around two main axes, networking (encouraging exchanges between young people from different clinical specialties) and the promotion of young infectious diseases professionals among their peers.

(i) For the first axis, networking axis, the ReJMiC organized several receptions with the aim of bringing together young clinical microbiologists and young clinical infectiologists of the ReJIF (Réseau des Jeunes Infectiologues Français, Network of young French infectiologists). Among these receptions, the event "serious game" combining pedagogy and networking (Ghelfenstein-Ferreira et al. 2021). During informal events as previously cited, young people from different backgrounds can meet and get to know each other, thus creating their first professional network. In addition, to encourage contacts, the ReJMiC and the other young networks (ReJIF, Trainee association of ESCMID, JePPRI [Jeunes Professionnels de la Prévention du Risque Infectieux, Young Professionals in Infectious Risk Prevention]) invite each other to their respective congress. Finally, it is important for young infectious disease specialists to learn about the work of their clinical microbiology colleagues during their studies. To this end, ReJMiC has created a list of available microbiology laboratory that can accommodate young infectious disease specialists.

(ii) The second axis concerns the promotion of young clinical microbiologists among their peers. To this end, ReJMiC is actively working on organizing conference sessions specifically for young people, in collaboration with the ReJIF.”

2. Page 4, line 118:

“In 2021, Dellière and colleagues published a collaborative opinion paper between the Trainee Association of European Society of Clinical Microbiology and Infectious Diseases (ESCMID) and four national trainee networks including the Young Microbiologists section. They discuss their motivations for building networks, the importance of these networks and offer guidance for their creation and sustainability based on their extensive experience (defining the target audience and the objectives of the network, approaching national societies even in specific disciplines, built a steering committee, communications…) (1). Beyond the challenge of networking creation, this commentary emphasizes the benefits of interaction between young fundamental and clinical microbiologists in a scientific world in perpetual modification. To conclude, we have shared our experience of the creation of the French Young Microbiologists networks, hosted by the SFM, and laid special emphasis on the challenges faced and the benefits reaped during the COVID-19 pandemic. To conclude, we hope that our commentary will encourage young microbiologists from other societies, and other countries, to take a similar approach to highlight young microbiologists work and help them develop a perennial network essential to promising young researchers for a valuable service of science.”

Response to Reviewer 2

This commentary has the exciting title of Bringing clinical and fundamental young microbiologists together. And what an outstanding platform for early career networking, especially during a very difficult period of isolation. However, I was hoping to read more concrete examples of outcomes that arose from bringing these two groups of young microbiologists together. For example, have any of these networking events resulted in new cross-disciplinary collaborations, manuscripts, grants, training opportunities, etc? I appreciate that it may be too soon to have data on such things, given that the group was only formed in 2018. In that case, were there specific activities working toward any of these goals. What would a 10 year successful young microbiologist chapter look like in terms of tangible outcomes? Do you think this should be expanded to other Microbiology Societies, if it's not already (is it?)? What lessons would you share/advice would you give/goals would you suggest to leaders of new chapters?

We thank the reviewer 2 for these comments. We added various examples of outcomes that arose from bringing our two groups of young microbiologists together (ReJMiF and ReJMiC) (see above). We also removed the abstract and modified grammatical suggestions.

1. Page 3, line 83:

“The ReJMiC actively collaborates with other networks of young infectious diseases professionals in France and in Europe. The common projects are articulated around two main axes, networking (encouraging exchanges between young people from different clinical specialties) and the promotion of young infectious diseases professionals among their peers.

(i) For the first axis, networking axis, the ReJMiC organized several receptions with the aim of bringing together young clinical microbiologists and young clinical infectiologists of the ReJIF (Réseau des Jeunes Infectiologues Français, Network of young French infectiologists). Among these receptions, the event "serious game" combining pedagogy and networking (Ghelfenstein-Ferreira et al. 2021). During informal events as previously cited, young people from different backgrounds can meet and get to know each other, thus creating their first professional network. In addition, to encourage contacts, the ReJMiC and the other young networks (ReJIF, Trainee association of ESCMID, JePPRI [Jeunes Professionnels de la Prévention du Risque Infectieux, Young Professionals in Infectious Risk Prevention]) invite each other to their respective congress. Finally, it is important for young infectious disease specialists to learn about the work of their clinical microbiology colleagues during their studies. To this end, ReJMiC has created a list of available microbiology laboratory that can accommodate young infectious disease specialists.

(ii) The second axis concerns the promotion of young clinical microbiologists among their peers. To this end, ReJMiC is actively working on organizing conference sessions specifically for young people, in collaboration with the ReJIF.”

2. Page 4, line 118:

“In 2021, Dellière and colleagues published a collaborative opinion paper between the Trainee Association of European Society of Clinical Microbiology and Infectious Diseases (ESCMID) and four national trainee networks including the Young Microbiologists section. They discuss their motivations for building networks, the importance of these networks and offer guidance for their creation and sustainability based on their extensive experience (defining the target audience and the objectives of the network, approaching national societies even in specific disciplines, built a steering committee, communications…) (1). Beyond the challenge of networking creation, this commentary emphasizes the benefits of interaction between young fundamental and clinical microbiologists in a scientific world in perpetual modification. To conclude, we have shared our experience of the creation of the French Young Microbiologists networks, hosted by the SFM, and laid special emphasis on the challenges faced and the benefits reaped during the COVID-19 pandemic. To conclude, we hope that our commentary will encourage young microbiologists from other societies, and other countries, to take a similar approach to highlight young microbiologists work and help them develop a perennial network essential to promising young researchers for a valuable service of science.”

**Reviews of manuscript FEMSMC-2022-007.r1**

**Reviewer 1**

**Recommendation: Accept**

The article looks good--I could find only three minor grammar issues that need to be corrected:

Line 92, “combining pedagogy and networking” should be "combined pedagogy and networking"

Line 100, “available microbiology laboratory” should be "available microbiology laboratories"

Line 133, ” microbiologists work” should be "microbiologists’ work"

**Reviewer 2**

**Recommendation: Accept**

Line 128, capitalize we
